# Associations between Minority Health Social Vulnerability Index Scores, Rurality, and Histoplasmosis Incidence, 8 US States

**DOI:** 10.3201/eid3010.231700

**Published:** 2024-10

**Authors:** Dallas J. Smith, Malavika Rajeev, Kristina Boyd, Kaitlin Benedict, Ian Hennessee, Laura Rothfeldt, Connie Austin, Mary-Elizabeth Steppig, Dimple Patel, Rebecca Reik, Malia Ireland, Judi Sedivy, Suzanne Gibbons-Burgener, Renee M. Calanan, Samantha L. Williams, Sarah Rockhill, Mitsuru Toda

**Affiliations:** Centers for Disease Control and Prevention, Atlanta, Georgia, USA (D.J. Smith, M. Rajeev, K. Boyd, K. Benedict, I. Hennessee, R.M. Calanan, S.L. Williams, S. Rockhill, M. Toda);; Arkansas Department of Health, Little Rock, Arkansas, USA (L. Rothfeldt);; Illinois Department of Public Health, Springfield, Illinois, USA (C. Austin);; Indiana Department of Health, Indianapolis, Indiana, USA (M.-E. Steppig);; Kentucky Department of Public Health, Frankfort, Kentucky, USA (D. Patel);; Michigan Department of Health and Human Services, Lansing, Michigan, USA (R. Reik);; Minnesota Department of Health, St. Paul, Minnesota, USA (M. Ireland);; Pennsylvania Department of Health, Harrisburg, Pennsylvania, USA (J. Sedivy);; Wisconsin Department of Health Services, Madison, Wisconsin, USA (S. Gibbons-Burgener)

**Keywords:** histoplasmosis, fungi, dimorphic fungi, social vulnerability index, health equity, rural health, healthcare access, healthcare infrastructure, United States

## Abstract

To explore associations between histoplasmosis and race and ethnicity, socioeconomic status, and rurality, we conducted an in-depth analysis of social determinants of health and histoplasmosis in 8 US states. Using the Minority Health Social Vulnerability Index (MH SVI), we analyzed county-level histoplasmosis incidence (cases/100,000 population) from the 8 states by applying generalized linear mixed hurdle models. We found that histoplasmosis incidence was higher in counties with limited healthcare infrastructure and access as measured by the MH SVI and in more rural counties. Other social determinants of health measured by the MH SVI tool either were not significantly or were inconsistently associated with histoplasmosis incidence. Increased awareness of histoplasmosis, more accessible diagnostic tests, and investment in rural health services could address histoplasmosis-related health disparities.

Histoplasmosis is an environmentally acquired fungal disease caused by *Histoplasma* species. In the United States, *Histoplasma* most commonly lives in central and eastern states, but infections also have been acquired outside of those areas (*1*,*2*). Globally, *Histoplasma* has been acquired in Latin America, Central Africa, and Southeast Asia but probably has worldwide distribution (*3*). Transmission involves inhalation of fungal spores from the environment; the incubation period is 3–17 days, and the primary clinical manifestation is pulmonary disease, although dissemination can occur (*4*,*5*). Commonly reported exposures include handling plant matter, disturbing material with bird or bat droppings, and cleaning, remodeling, or tearing down buildings (*6*,*7*). Persons who live in rural areas appear to be disproportionately affected by histoplasmosis, although outbreaks also can occur in urban settings (*6*–*8*).

Health disparities have been identified for fungal diseases in general, although additional analyses are needed to explore the underlying causes (*8*,*9*). Those disparities probably are related to environmental, behavioral, demographic, occupational, and socioeconomic factors. For histoplasmosis, previous reports have shown that men and boys and persons 41–80 years of age are more likely to have the disease diagnosed. Although incidence rates have been found to be similar across racial and ethnic categories (*1*,*6*), some studies of hospitalization data have found lower histoplasmosis-associated hospitalization rates among White patients (*10*). A previous study showed higher histoplasmosis hospitalization rates among non-Hispanic White patients and more histoplasmosis diagnoses among adult, low-income, and rural patients; however, analyses of associations between social, structural, and geographic factors that affect health and histoplasmosis incidence are lacking (*8*). Understanding such associations can inform educational outreach and other public health interventions to prevent illness and death from histoplasmosis in communities at higher risk.

To explore associations between histoplasmosis incidence, social determinants of health and rurality, we analyzed county-level histoplasmosis incidence from 8 US states reporting histoplasmosis to public health authorities. For this study, we used the Minority Health Social Vulnerability Index (MH SVI) and the National Center for Health Statistics (NCHS) urban–rural classification scheme.

## Methods

### Histoplasmosis Incidence

During 2011–2014 and 2019, a total of 8 US states (Arkansas, Illinois, Indiana, Kentucky, Michigan, Minnesota, Pennsylvania, Wisconsin) encompassing 698 counties reported county-level histoplasmosis case counts directly to the Centers for Disease Control and Prevention (CDC) Mycotic Diseases Branch (housed in the Division of Foodborne, Waterborne and Environmental Diseases, National Center for Emerging and Zoonotic Infectious Diseases). In 2020, those states reported histoplasmosis data to the National Notifiable Disease Surveillance System. Data from 2015–2018 were not reported to CDC. We calculated 6-year cumulative county-level incidence and 95% CIs by using county of residence and US Census population estimates across the years included in the study (*11*).

### Minority Health Social Vulnerability Index

The MH SVI, launched in 2021, was developed by the US Department of Health and Human Services Office of Minority Health and CDC as an expanded version of CDC’s Social Vulnerability Index. The MH SVI organizes 34 county-level social and structural factors that affect health into 6 distinct themes: socioeconomic status, household composition and disability, minority status and language, housing type and transportation, healthcare infrastructure and access, and medical vulnerability (*12*). This tool supports identification of racial and ethnicity minority communities that may be disproportionately affected by public health threats. MH SVI theme scores are interpreted as percentile rankings and expressed as decimals from 0 to 1; higher scores represent more vulnerable counties. We applied the methods for calculating and ranking the national MH SVI scores to obtain a regional score for each theme by including only data from the 8 states submitting histoplasmosis data (Appendix Figure 1). We also calculated MH SVI scores stratified by urban–rural classifications.

### NCHS Urban–Rural Classification Scheme

The 2013 NCHS urban–rural classification scheme categorizes counties based on 6 levels of urbanization and is useful for assessing and monitoring health differences between counties (*13*). Level 1 (large central metropolitan) is the most urban, whereas level 6 (noncore) is the most rural. We condensed the NCHS classifications into 3 categories by combining large central metropolitan (level 1 [10 counties]) and large fringe metropolitan (level 2 [89 counties]), medium metropolitan (level 3 [71 counties]) and small metropolitan (level 4 [164 counties]), and micropolitan (level 5 [164 counties]) and noncore (level 6 [279 counties]). We collapsed the classifications on the basis of sparse data in the most urban category and a similar study of Social Vulnerability Index metrics and rurality (*14*).

### Statistical Analyses

We used generalized linear mixed hurdle models to model 2 outcomes: the probability of observing >1 case (the zero-inflated component of the model [i.e., logistic regression]), and 6-year cumulative case counts at the county level (conditional component of the model [i.e., truncated Poisson regression]). We used a hurdle model to account for excess zeros in the data (i.e., counties that did not report any histoplasmosis cases) that might be attributable to sampling processes (e.g., limited surveillance or chance) (*15*). We summed case counts and population estimates across the 6 years (2011–2014 and 2019–2020) of available data. We ran models with counties categorized into low (referent), medium, and high-ranking tertiles for each MH SVI theme. As a sensitivity analysis, we also ran models with the MH SVI themes as continuous covariates. We included a state random intercept to account for state-specific differences in incidence and case detection and used the 3-category NCHS urban–rural classification to account for associations between rurality and histoplasmosis incidence. We included all covariates in both the zero-inflated and conditional components of the model. In addition, we included county population size as an offset in the conditional component and as a covariate (centered and scaled) in the zero-inflated component. To account for potential confounding between rurality and MH SVI metrics, we ran an analysis stratified by urban–rural classification, running models for 443 micropolitan and noncore counties, 156 medium and small metropolitan counties, and 99 large metropolitan counties by using stratified MH SVI measures (recalculated for the subset of counties in each stratum).

We conducted analyses by using R version 4.2.2 (*16*). We implemented all models by using the R package glmmTMB (*15*) and used the R package DHARMa for model residual diagnostics, testing for uniformity of residuals, outlier predictions, and over- and under-dispersion (*17*).

## Results

**Figure 1:**
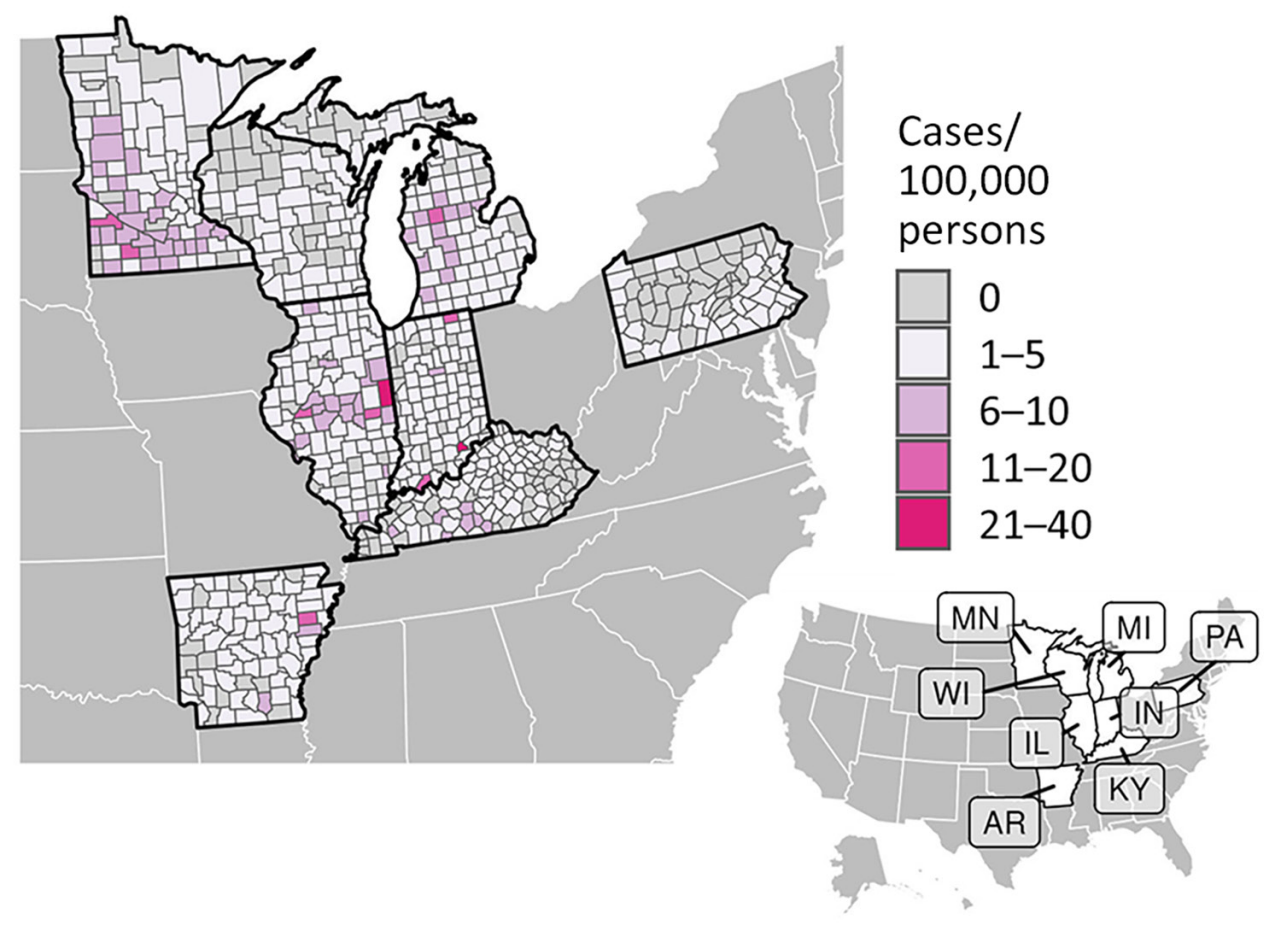
County-level histoplasmosis incidence (cases/100,000 persons) in 8 US states for which data were available, 2011–2014 and 2019–2020. Inset map indicates the 8 states.

**Figure 2:**
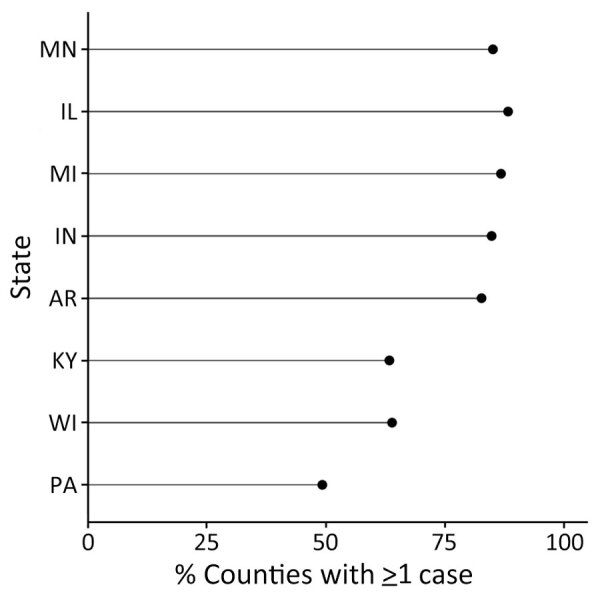
Percentage of counties in each state with >1 reported case of histoplasmosis in 8 US states for which data were available, 2011–2014 and 2019–2020.

**Figure 3:**
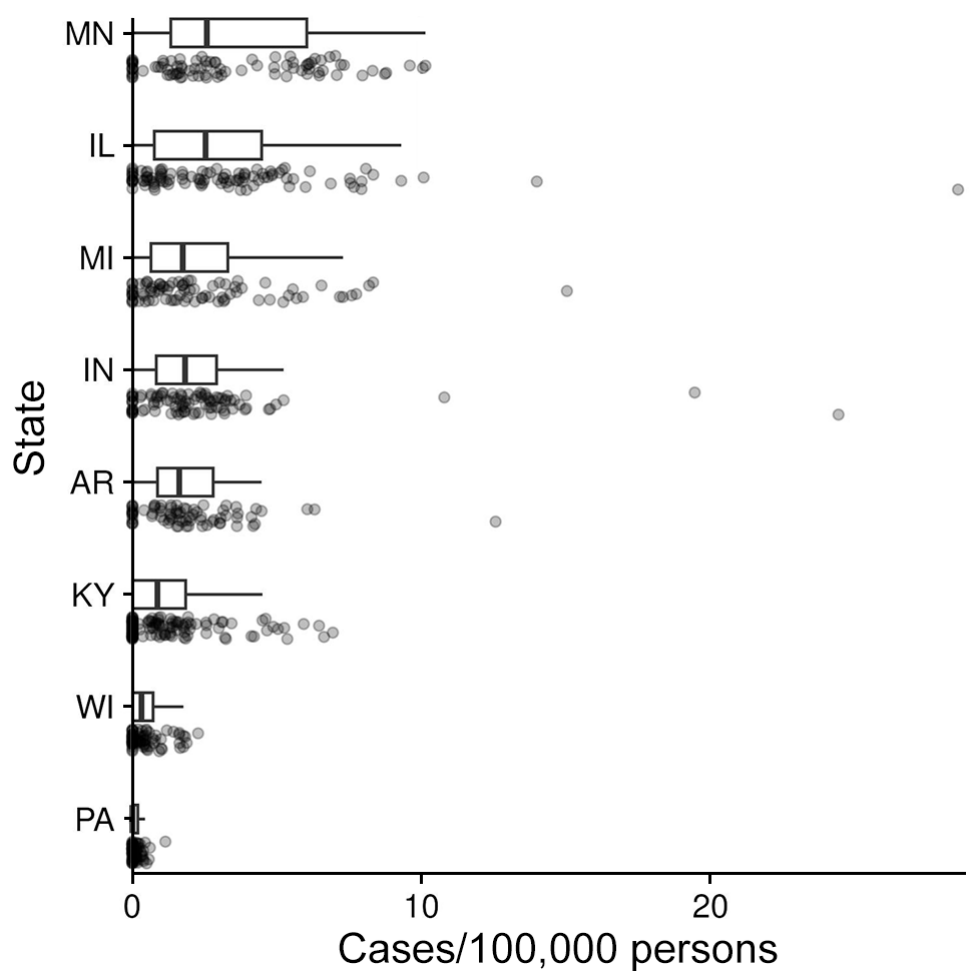
Distribution of county-level incidence (ordered from highest mean incidence to lowest mean incidence) in 8 US states for which data were available, 2011–2014 and 2019–2020. Boxplots show the medians (vertical black lines), interquartile ranges (box left and right ends), and range +1.5 × interquartile range (error bars); the points show the raw data.

During 2011–2014 and 2019–2020, a total of 4,854 histoplasmosis cases were reported in the 8 states included in this study. Of 698 total counties in the 8 states, 531 reported >1 case. For counties reporting >1 case, incidence ranged from 0.06 to 28.57 cases/100,000 persons. Both the proportion of counties with a case and the incidence varied by state (Figure 1); Minnesota had the highest average county-level incidence and Pennsylvania the lowest (Figures 2, 3). Median annual incidence was generally consistent across years, and of the counties reporting >1 case, most reported >1 across multiple years (73%) (Appendix Figure 2).

**Figure 4:**
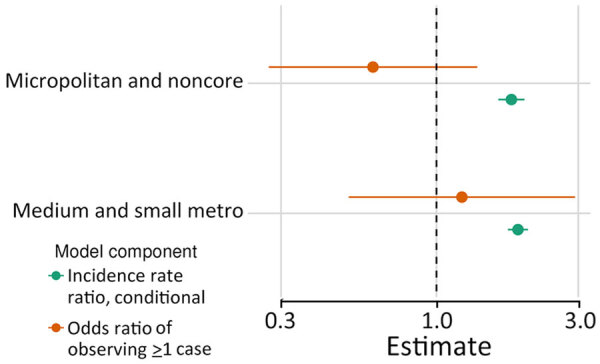
Associations between rurality and histoplasmosis incidence for counties reporting >1 case in 8 US states for which data were available, 2011–2014 and 2019–2020. Incidence rate ratios for conditional component (green) and odds ratios for the probability of observing >1 case in the zero-inflated component (orange) are shown 95% CIs (error bars) by county rural classification; reference group is large metropolitan counties.

**Figure 5:**
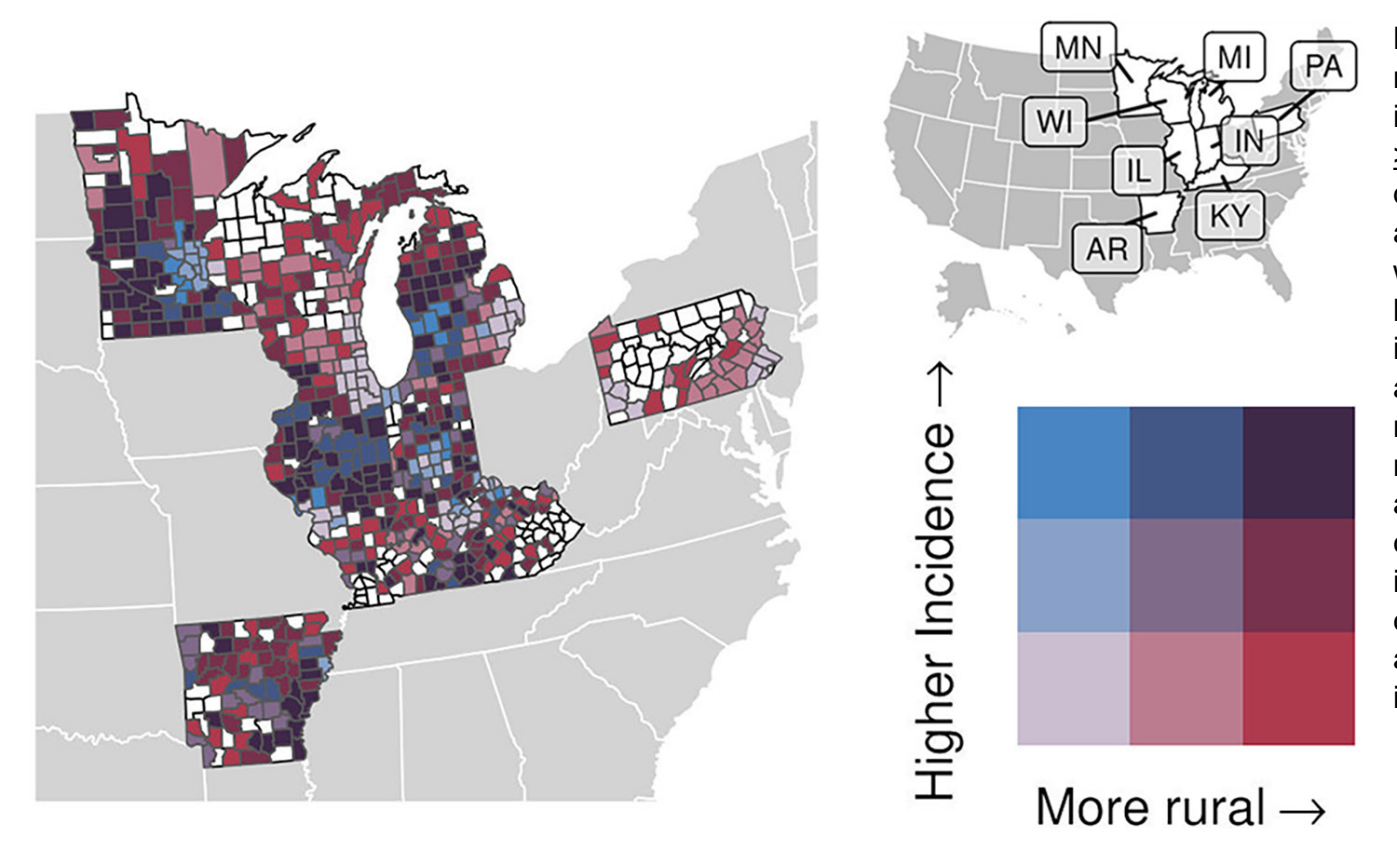
Associations between rurality and histoplasmosis incidence for counties reporting >1 case in 8 US states for which data were available, 2011–2014 and 2019–2020. For counties with >1 case of histoplasmosis, bivariate map shows county incidence (split into low-, mid-, and high-incidence tertiles) versus rurality (micropolitan and noncore, medium and small metropolitan, and large metropolitan counties); colors indicate the combination of incidence-rurality levels for each county. Counties without a case are shown in white. Inset map indicates names of the 8 states.

For counties reporting >1 case of histoplasmosis, all MH SVI themes were significantly associated with incidence in the count model (Appendix Table). MH SVI scores for the socioeconomic status and minority status and language themes were negatively associated with incidence; counties in the medium and high tertiles for those themes (more vulnerable counties) had significantly lower incidence compared with counties in the low tertile. In contrast, counties with higher vulnerability scores for the household composition and disability, healthcare infrastructure and access, and medical vulnerability themes had significantly higher incidence compared with low vulnerability counties. Counties classified as micropolitan and noncore and small and medium metropolitan had significantly higher incidence compared with the most urban counties (large metropolitan counties) (Figures 4, 5; Appendix Table).

**Figure 6:**
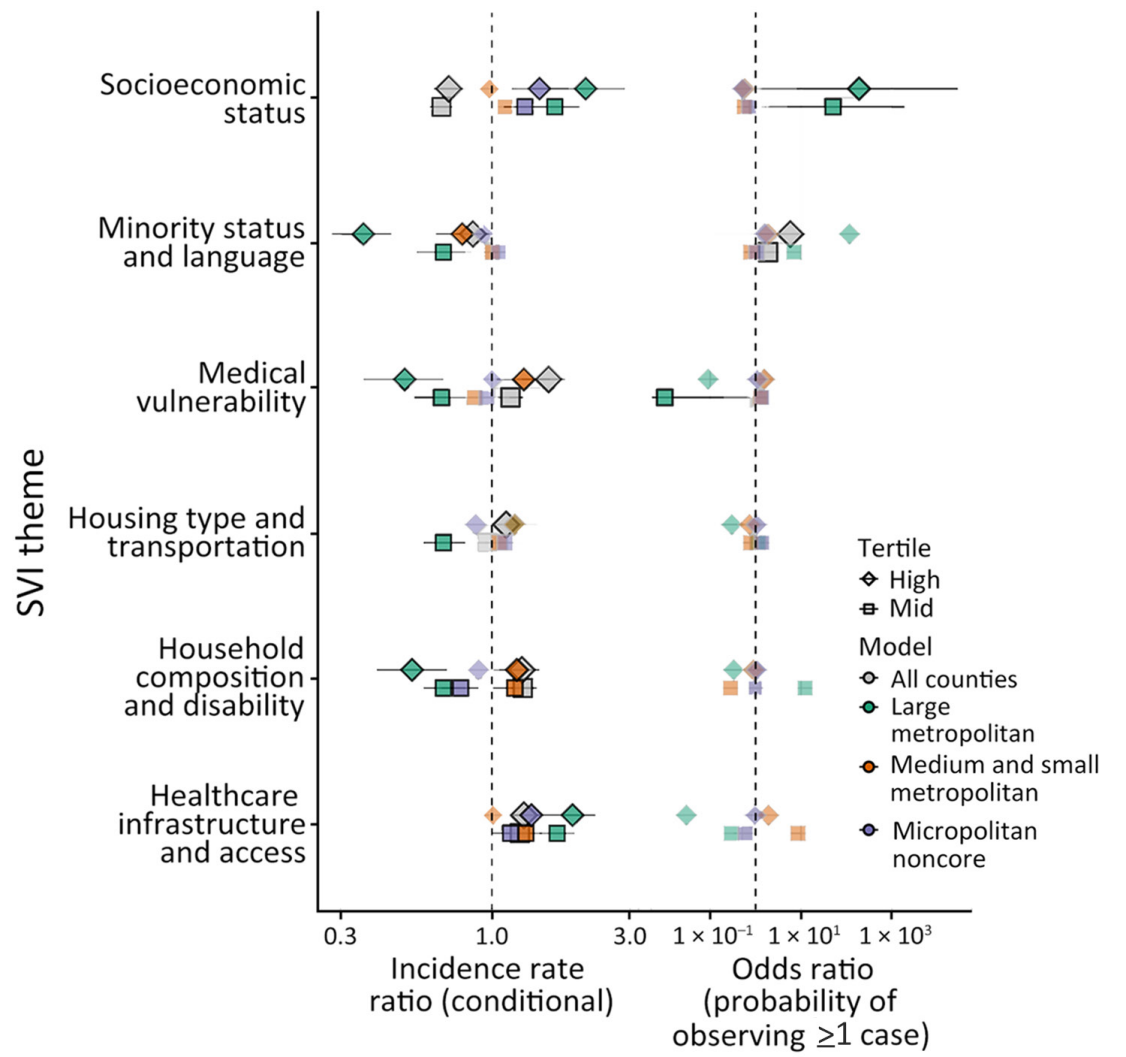
Model effect estimates for association of histoplasmosis incidence with MH SVI themes in 8 US states for which data were available, 2011–2014 and 2019–2020. MH SVI theme scores are interpreted as percentiles; higher scores represent more vulnerable counties. Left column shows incidence rate ratios for the conditional component; right column shows odds ratios for the probability of observing a case in the zero-inflated component. Error bars indicate 95% CIs. Shapes indicate the tertile (mid-tertile or high-tertile, with low-tertile as the reference level), and color indicates the model (model with all counties vs. those stratified by rural classification). Statistically significant effects are indicated by a black outline and increased opacity of points. MH SVI, Minority Health Social Vulnerability Index.

Because rurality was significantly associated with incidence and the distribution of MH SVI scores, we ran models with counties stratified by rurality and recalculated MH SVI for the subset of counties in each urban–rural class to mitigate these associations (Appendix Figure 3). Many of the associations observed between MH SVI themes and incidence in the unstratified model were inconsistent in direction and statistical significance in the stratified models (Figure 3). Healthcare infrastructure and access was the only theme that had a consistent association and significance across urban–rural strata; counties with mid-vulnerability, high-vulnerability, or both had significantly higher incidence than low-vulnerability counties (Figure 6). The positive association between higher minority status and language vulnerability score and histoplasmosis incidence only remained statistically significant in the model with large metropolitan counties. In the unstratified model, more vulnerable counties for the socioeconomic status theme had lower incidence; however, the direction of that association flipped for micropolitan and noncore and large metropolitan counties and was not statistically significant for small and medium metropolitan counties (Figure 6). The association between the socioeconomic status theme and incidence was sensitive to our classification of counties into MH SVI tertiles; this association was not statistically significant in models with MH SVI themes as continuous covariates (Appendix Figure 4).

Minority status and language was the only MH SVI theme significantly associated with the probability of observing >1 case at the county level in the zero-inflated model (Appendix Table). County population size was associated with higher probability of observing a case, but rural classification did not have a statistically significant effect (Appendix Table, Figure 4). Rural classification, population size, and the minority status and language theme were correlated (Appendix Figure 5), and when we stratified by urban–rural classification, the association between minority status and language and observing a case was not significant (Figure 6). For the model with large metropolitan counties, socioeconomic status and medical vulnerability themes were significantly associated with case observation, but with only 99 counties in that category, the model also had the highest uncertainty (Figure 6; Appendix Figure 4).

Our key findings were not dependent on classification of counties into vulnerability tertiles; models with MH SVI themes as continuous covariates showed the same results for rurality and the healthcare access and infrastructure theme as the tertile models and overall were mostly congruent in the estimated associations and statistical significance for all other covariates (Appendix Figure 4). Estimates for state intercepts were also consistent across all models (Appendix Figure 6); however, much of the variation in incidence could not be explained by associations with MH SVI themes, rurality, or state-specific differences in incidence (Appendix Figure 7). Although models were robust when tested for outlier predictions, overdispersion, and underdispersion, the models with all counties and micropolitan and noncore counties did have significant nonuniformity of residuals (Appendix Figure 8), indicating potential issues with model fit.

## Discussion

We found that histoplasmosis incidence was higher in counties with limited healthcare infrastructure and access as measured by the MH SVI theme and in more rural counties. In the full model, many of the other themes also were significantly associated with histoplasmosis incidence; however, these effect sizes were inconsistent in statistical significance and direction of the effect when stratified by urban–rural classification (i.e., household composition and disability and medical vulnerability). By using a hurdle model, we were able to model case observation separately from incidence and found that MH SVI themes were poor predictors of whether a county reported >1 histoplasmosis case during the study period.

Counties in the highest vulnerability tertile for the healthcare infrastructure and access theme had higher histoplasmosis incidence rates than did low-vulnerability counties. Counties in the highest vulnerability tertile probably have less access to hospitals, urgent care clinics, pharmacies, and primary-care physicians, perhaps making a diagnosis of histoplasmosis more difficult to obtain, which means histoplasmosis incidence probably is underestimated in these counties. Previous reports have shown that many patients with histoplasmosis experience >3 missed opportunities for diagnosis, leading to diagnostic delays of >3 weeks (*6*,*18*). Most histoplasmosis diagnoses are made by pulmonologists and infectious disease physicians (*6*). Access to such specialized providers is limited in the highly vulnerable communities. Considering there are more hospital closures than openings and few urgent care centers, especially in rural areas, it is critical for primary-care providers to consider histoplasmosis in patients who live in or have traveled to histoplasmosis-endemic areas who have compatible signs and symptoms (e.g., fever, cough, fatigue, chills, headache, chest pain, body aches) without improvement after empiric antibacterial medications (*19*,*20*). Extensive exposure to bird or bat droppings, a chest radiograph demonstrating new nodules or lymphadenopathy consistent with histoplasmosis, or an epidemiologic link to a histoplasmosis outbreak may be obtained from a patient’s medical history when determining whether to test for histoplasmosis (*7*,*21*).

Equipping healthcare facilities with appropriate diagnostic tools might reduce histoplasmosis disparities related to healthcare infrastructure and access. Current diagnostic options are limited and can have long turnaround times, and test results can be difficult to interpret (*5*). For example, certain *Histoplasma* antigen assays are not commercially available; the assays that are available may be cost-prohibitive in rural healthcare facilities. Multiple and repeated diagnostic tests may be needed for an accurate diagnosis (*22*,*23*). Healthcare systems could use clinical diagnostic guidance for histoplasmosis to prioritize appropriate testing to manage resources and diagnostic access issues (*3*).

Promising diagnostic technologies may be able to revolutionize testing for histoplasmosis to prevent delayed diagnoses and misdiagnoses. *Histoplasma* antigen lateral flow assays (LFAs) show promise as point-of-care screening tests with high sensitivity (although further testing is needed in persons not living with HIV), are noninvasive, and have quick turnaround times (*24*–*28*). Encouragingly, multiple LFAs are undergoing diagnostic performance evaluation (*28*). LFAs could help mitigate vulnerabilities related to poor healthcare infrastructure and access by expediting diagnosis and subsequent treatment. Government-supported flexibilities for telemedicine and ordering diagnostic tests, like those implemented during the COVID-19 pandemic, especially in high-vulnerability counties, could improve access to care for patients with histoplasmosis (*29*).

Consistent with findings from previous studies, we found that the higher incidence rates in rural areas might be related to activities and occupations resulting in exposure to *Histoplasma* (*6*). Construction, excavation, agriculture, forestry, and hunting occupations have been linked to increased risks for acquiring histoplasmosis (*30*). Gardening, landscaping, or other handling of plant matter (48%); digging in soil (37%); and handling bird or bat droppings (24%) were identified as common exposures in patients with histoplasmosis in a recent surveillance report (*6*). Those occupations and activities might be more prevalent in rural areas. For workers at risk for histoplasmosis, appropriate prevention methods are critical (*30*). Interventions could include removing bats or birds from buildings, limiting dust exposure, communicating hazards, educating on histoplasmosis, and encouraging use of National Institute for Occupational Safety and Health–approved respirators for high-risk activities. We found that even among rural counties, those with higher vulnerability in the healthcare access and infrastructure theme had higher incidence of histoplasmosis, suggesting that interactions between exposure factors in rural areas and social and structural vulnerabilities may compound the risk for histoplasmosis.

Neither MH SVI themes nor rurality were strong predictors of which counties reported any histoplasmosis cases. County- and state-level differences in case detection and reporting and other factors that influence baseline risk (e.g., underlying conditions and environmental conditions) might be more predictive. Histoplasmosis surveillance in the United States is limited in terms of the number of states reporting, the data collected about cases, and the fact that histoplasmosis is not a nationally notifiable disease. A national case definition for histoplasmosis was established in 2017; state public health authorities used varying case definitions during 2011–2014 (*31*), making intrastate and interstate comparisons of histoplasmosis incidence difficult.

The medical vulnerability theme captures certain underlying conditions but does not capture severity of the condition (e.g., poorly controlled diabetes) and is not comprehensive; for example, autoimmune disease is not included, yet it was the most common underlying condition reported among patients with histoplasmosis in an enhanced surveillance study (*6*). *Histoplasma* is not distributed uniformly among counties, and our analysis was not able to account for hot spots or foci of *Histoplasma*, which are often associated with accumulated bird or bat droppings (*7*). Other environmental conditions that are incompletely understood also may influence *Histoplasma* hot spots. Modeling efforts have begun to explore areas where *Histoplasma* may be more prevalent (e.g., using suitability scores based on preferred soil environments) (*32*,*33*). More research is needed in that area, but such models could account for geographic variation at a more granular level than our study. Other social determinants of health (e.g., occupation and working conditions) not measured by the MH SVI tool have been linked to histoplasmosis outbreaks and probably are associated with higher histoplasmosis incidence (*30*). In future analyses, additional determinants could be included to further explore factors related to histoplasmosis incidence and guide public health response (*34*).

One limitation of our analysis is that MH SVI measures are composite ranks and therefore only can be interpreted in relative terms and within the context of the locations represented in the analysis. We found that it was critical to recalculate indices for the subset of states analyzed and stratify by rurality when examining associations between themes and incidence, both of which are associated with rurality. Extrapolation to the entire United States may not be appropriate because only 8 states were included in this study. MH SVI metrics are snapshots rather than temporal measures and thus do not capture changes in the individual factors that may occur over the time. In addition, the NCHS urban–rural classification scheme is largely based on proximity to a large metropolitan area and therefore may not capture aspects of rurality that might be more strongly associated with histoplasmosis incidence. It does not directly correlate with population density or other factors, such as land use or occupational composition, which may be more directly related to exposure risk. Our analyses also were limited to data aggregated to the county level. Rurality, population density, access to services, racial and ethnic diversity, and other social factors can vary widely within counties, and our estimates could not include individual-level risk factors for histoplasmosis. Future studies could address some of these limitations by incorporating more specific measures of rurality and social vulnerability (e.g., land use or individual data elements within the MH SVI themes), higher resolution spatial data (e.g., at the census tract), or individual-level sociodemographic and behavioral data (e.g., insurance status, care-seeking behavior, and occupational or environmental exposures). Moreover, county-level case counts were based on county of residence. Acquisition of histoplasmosis can be related to activities or an occupation that may not take place within a patient’s county of residence; thus, county of residence may not always represent the location where the infection was acquired. Also, data on histoplasmosis outcome (e.g., illness, decreased quality of life, and death) and clinical manifestations (e.g., pulmonary or disseminated) were not available, but understanding the associations of those variables with social determinants of health could be critical to save lives and could be explored with enhanced histoplasmosis surveillance. Further, changes (e.g., in the economy and climate) may have occurred during 2011–2019 in the counties that were not captured in this analysis. We saw consistent incidence in states across years and counties reporting >1 case, but we cannot rule out the possibility of bias introduction by the gaps in data during 2015–2018.

In conclusion, our study found histoplasmosis incidence was higher in counties with limited healthcare infrastructure and access and in rural counties. Other social determinants of health measured by the MH SVI tool were not associated with histoplasmosis incidence. Increased awareness of histoplasmosis among healthcare providers and the public, implementation of prevention measures for occupational-related disease, point-of-care histoplasmosis diagnostic tools, and overall investment in rural health services are needed to help address histoplasmosis-related health disparities.

Appendix Additional information about associations between minority health social vulnerability index scores, rurality, and histoplasmosis incidence, eight US states.
