# Supplementary material for: Associations between Minority Health Social Vulnerability Index Scores, Rurality, and Histoplasmosis Incidence, 8 US States
Source: Emerg Infect Dis. 2024 Oct;30(10):2016–24. doi: 10.3201/eid3010.231700 (PMC11431912; doi:10.3201/eid3010.231700)
Supplement: Appendix — Additional information about associations between minority health social vulnerability index scores, rurality, and histoplasmosis incidence, eight US states. [file 23-1700-Techapp-s1.pdf]

Article DOI: https://doi.org/10.3201/eid3010.231700

Associations between Minority Health Social Vulnerability Index Scores, Rurality, and Histoplasmosis Incidence, Eight US States

Appendix

Appendix Table. Associations between the Minority Health Social Vulnerability Index (MH SVI) scores, rurality, and histoplasmosis incidence rates using a hurdle model — United States, 2011–2014, 2019–2020

	Histoplasmosis Crude Incidence (95% CI)			Count model: Adjusted Incidence Rate Ratios (IRR, 95% CI)*		Zero-inflated model: Adjusted Odds Ratios (OR, 95% CI)†	
MH SVI Theme‡	Low tertile (0–0.33)	Medium tertile (0.34–0.66)	High tertile (0.67–1)	Medium tertile vs. low tertile estimate	High tertile vs. low tertile estimate	Medium tertile vs. low tertile estimate	High tertile vs. low tertile estimate
Socioeconomic status	1.26 (1.21–1.32)	1.30 (1.24–1.36)	1.54 (1.45–1.64)	**0.67 (0.61–0.73)**	**0.71 (0.63–0.81)**	1.17 (0.58–2.35)	0.50 (0.21–1.20)
Household composition and disability	1.15 (1.10–1.20)	1.28 (1.21–1.35)	1.94 (1.84–2.05)	**1.28 (1.17–1.40)**	**1.27 (1.14–1.41)**	0.81 (0.47–1.39)	1.12 (0.60–2.10)
Minority status and language	2.10 (1.92–2.29)	1.70 (1.58–1.83)	1.21 (1.17–1.25)	0.93 (0.82–1.05)	**0.86 (0.76–0.97)**	**1.88 (1.17–2.99)**	**5.82 (2.74–12.36)**
Housing type and transportation	1.45 (1.37–1.53)	1.12 (1.06–1.19)	1.39 (1.34–1.45)	0.96 (0.88–1.05)	**1.12 (1.03–1.23)**	1.25 (0.74–2.10)	1.18 (0.67–2.09)
Health care infrastructure and access	1.12 (1.07–1.17)	1.45 (1.39–1.51)	1.73 (1.62–1.85)	**1.25 (1.17–1.35)**	**1.29 (1.18–1.42)**	0.79 (0.46–1.33)	0.81 (0.48–1.37)
Medical vulnerability	1.17 (1.13–1.22)	1.29 (1.23–1.36)	2.17 (2.03–2.31)	**1.16 (1.05–1.28)**	**1.57 (1.37–1.79)**	1.24 (0.64–2.39)	1.32 (0.56–3.11)
Rurality§	Large metropolitan	Medium or small metropolitan	Micropolitan or noncore	Medium or small metropolitan vs. large metropolitan estimate	Micropolitan and noncore vs. large metropolitan	Medium or small metropolitan vs. large metropolitan estimate	Micropolitan or noncore vs. large metropolitan
	0.99 (0.95–1.04)	1.5 (1.44–1.59)	1.93 (1.83–2.04)	**1.88 (1.74–2.03)**	**1.79 (1.62–1.98)**	1.22 (0.51–2.93)	0.61 (0.27–1.37)

*IRRs represent the effects of each MH SVI theme on histoplasmosis incidence as estimated from the hurdle model (with the four MH SVI themes and rurality as covariates and county population as an offset). Model is conditional on counties with at least one histoplasmosis case during the time period.
†ORs represent the effect of each MH SVI theme on the odds of counties having at least one histoplasmosis case, controlling for other variables.
‡MH SVI theme scores are interpreted as percentiles; higher scores represent more vulnerable counties.
§The NCHS classifications were condensed into three categories by combining large central metropolitan (Level 1) and large fringe metropolitan (Level 2), medium metropolitan (Level 3) and small metropolitan (Level 4), and micropolitan (Level 5) and non-core (Level 6).

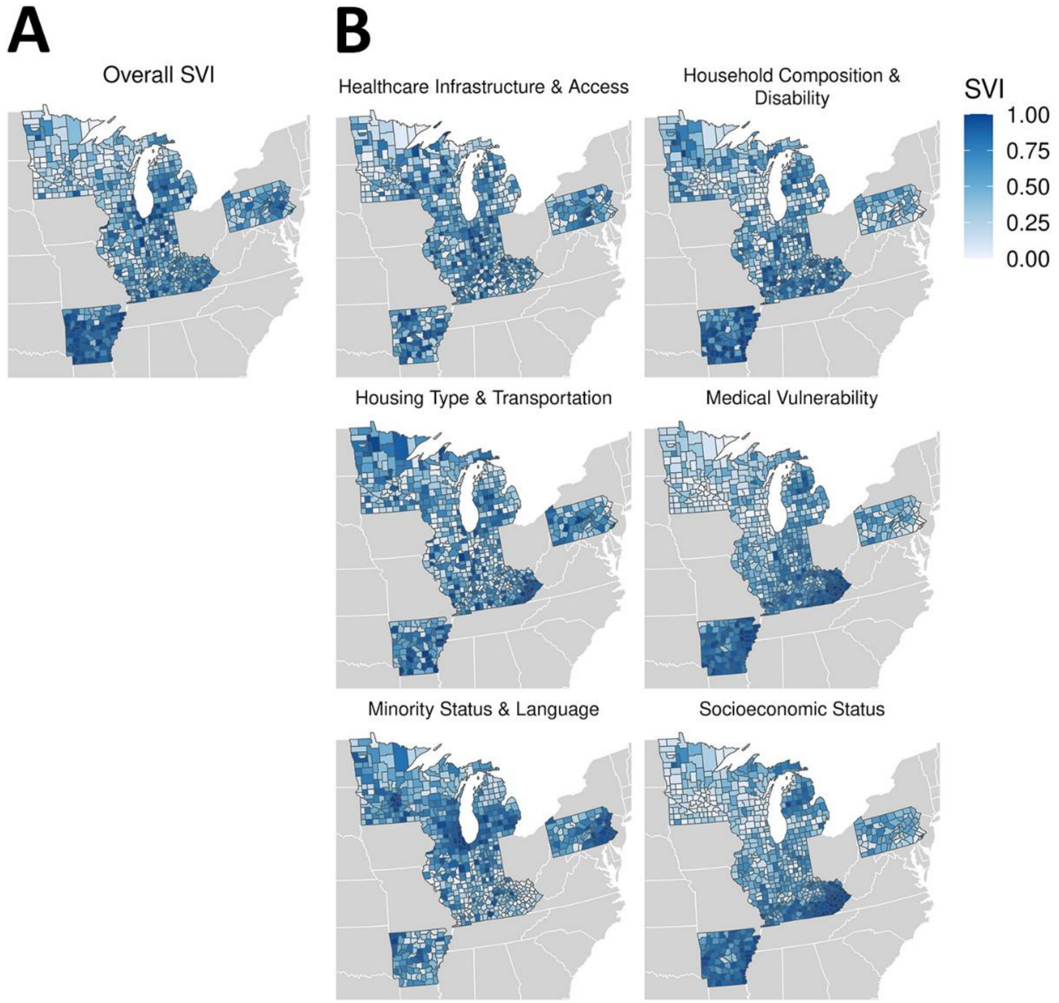

Appendix Figure 1. Map of estimated regional MH SVI across all counties in the eight states included in the study for A) overall MH SVI and B) all themes. Darker blue indicates higher vulnerability counties (higher index values).

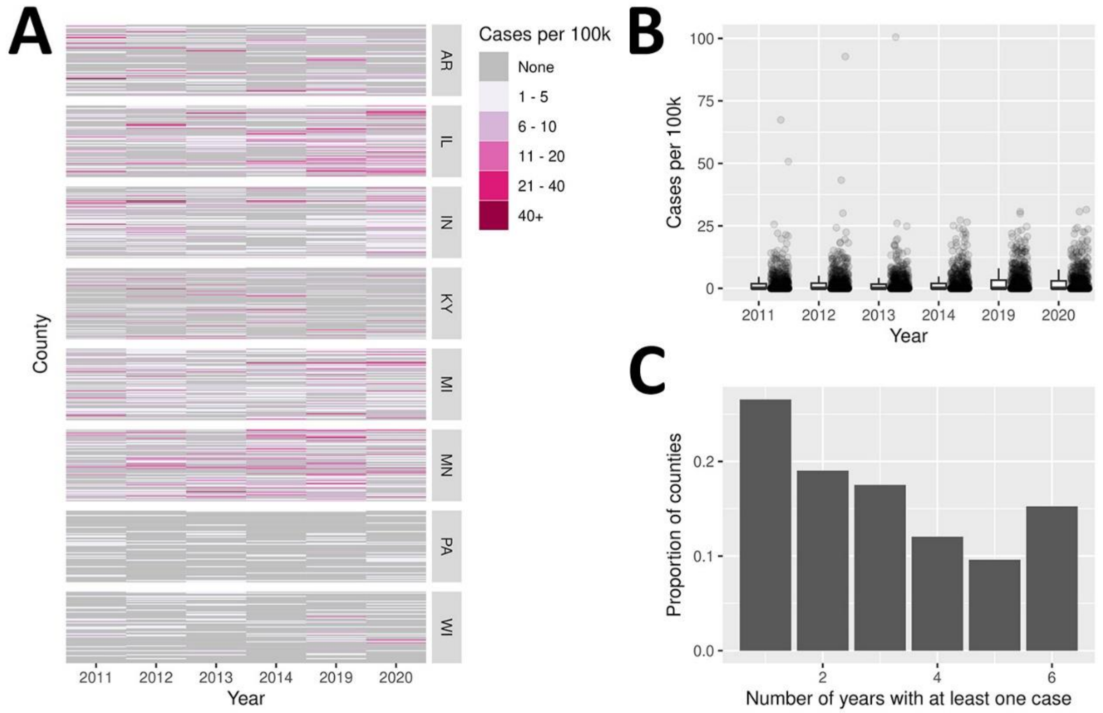

Appendix Figure 2. A) Annual incidence over the 6 years included in the study (2011 - 2014, 2019 - 2020). The y-axis are counties organized by state (panels) and x-axis the year, with the color indicating binned incidence per 100,000 persons. B) Annual incidence across all counties by year, with boxplot showing the median and interquartile range, and the points showing the full data. C) The number of years with a case for counties that reported at least one case (x-axis), and the proportion of counties that had a case for that number of years (y-axis).

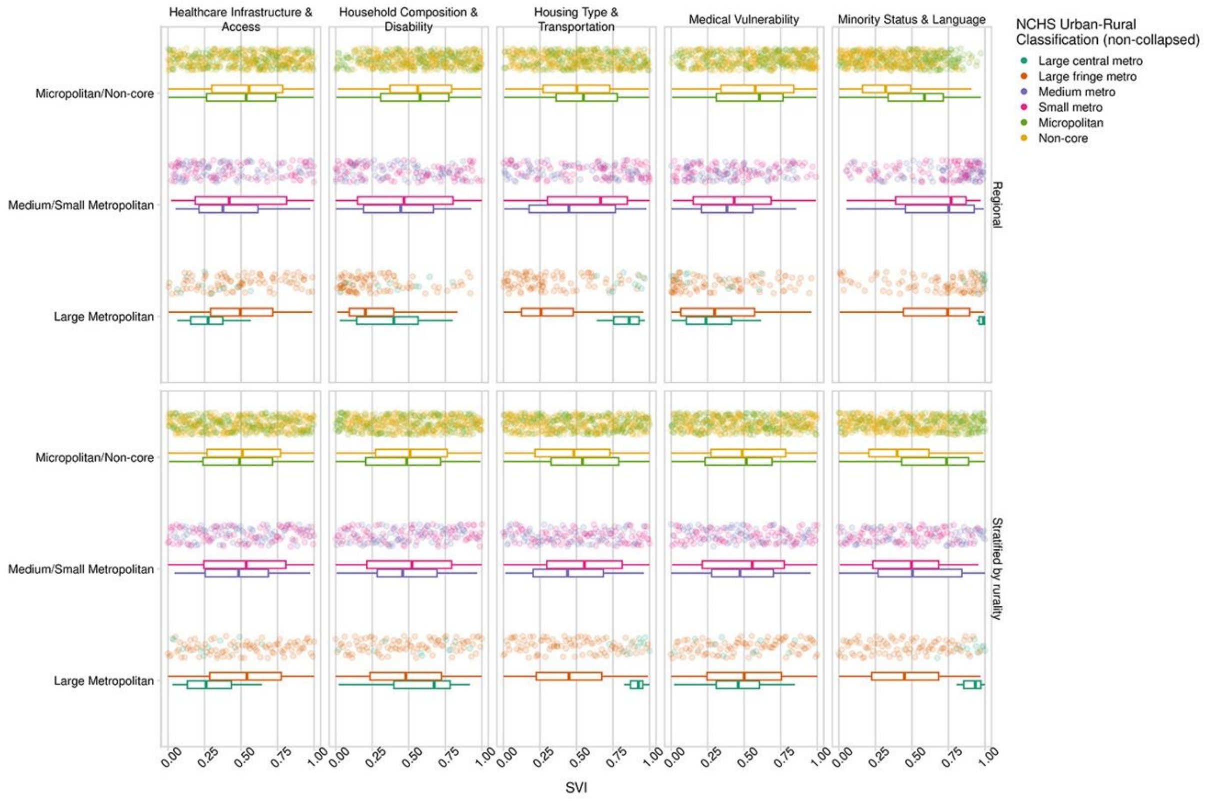

Appendix Figure 3. MH SVI (x-axis) for counties across rural classification. MH SVI theme scores are interpreted as percentiles; higher scores represent more vulnerable counties. The y-axis is the collapsed urban-rural classification used in the model and for stratification, and the colors indicate the non-collapsed NCHS urban-rural classification. The boxplots show the median and interquartile range, and the points show the raw data. The row panels are the regional MH SVI (top row, calculated across all counties in the eight states with data) and the stratified MH SVI (bottom row, calculated for the subset of counties in each of the collapsed rural classification). The column panels are each of the MH SVI themes included in the models.

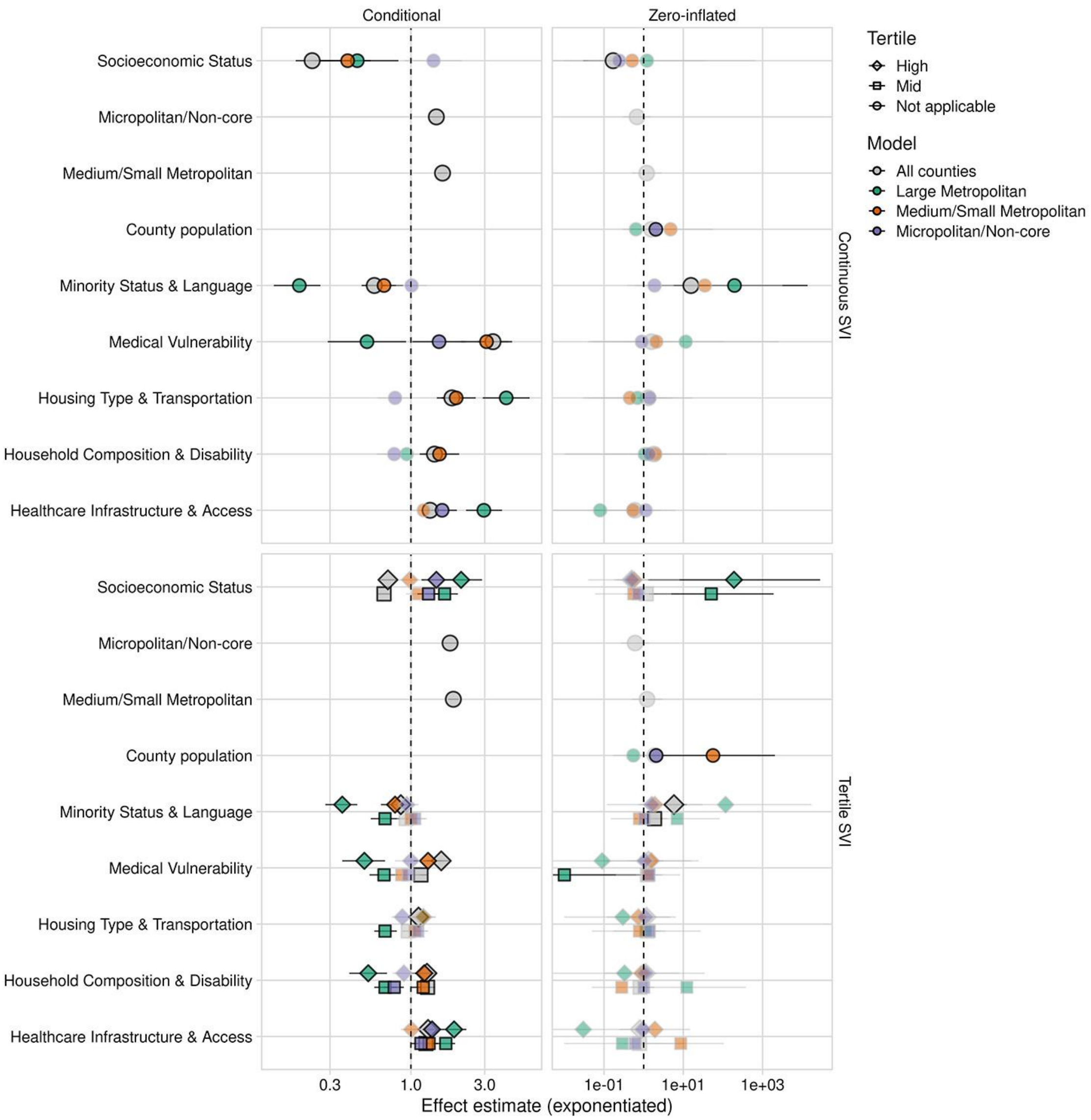

Appendix Figure 4. Effect estimates (exponentiated, x-axis) for all terms in models (y-axis). The row panels indicate how MH SVI themes were included in the model (either as continuous scores or tertiles), and the column panels are the conditional (predicting incidence) and the zero-inflated components (predicting the presence of at least one case) of the models. Shapes indicate the tertile (mid or high, with low tertile as the reference level; or not applicable for other terms that were not split by tertile), and color indicates the model (model with all counties vs. those stratified by rural classification). MH SVI theme scores are interpreted as percentiles; higher scores represent more vulnerable counties. Statistically significant effects are indicated by a black outline and increase opacity of points.

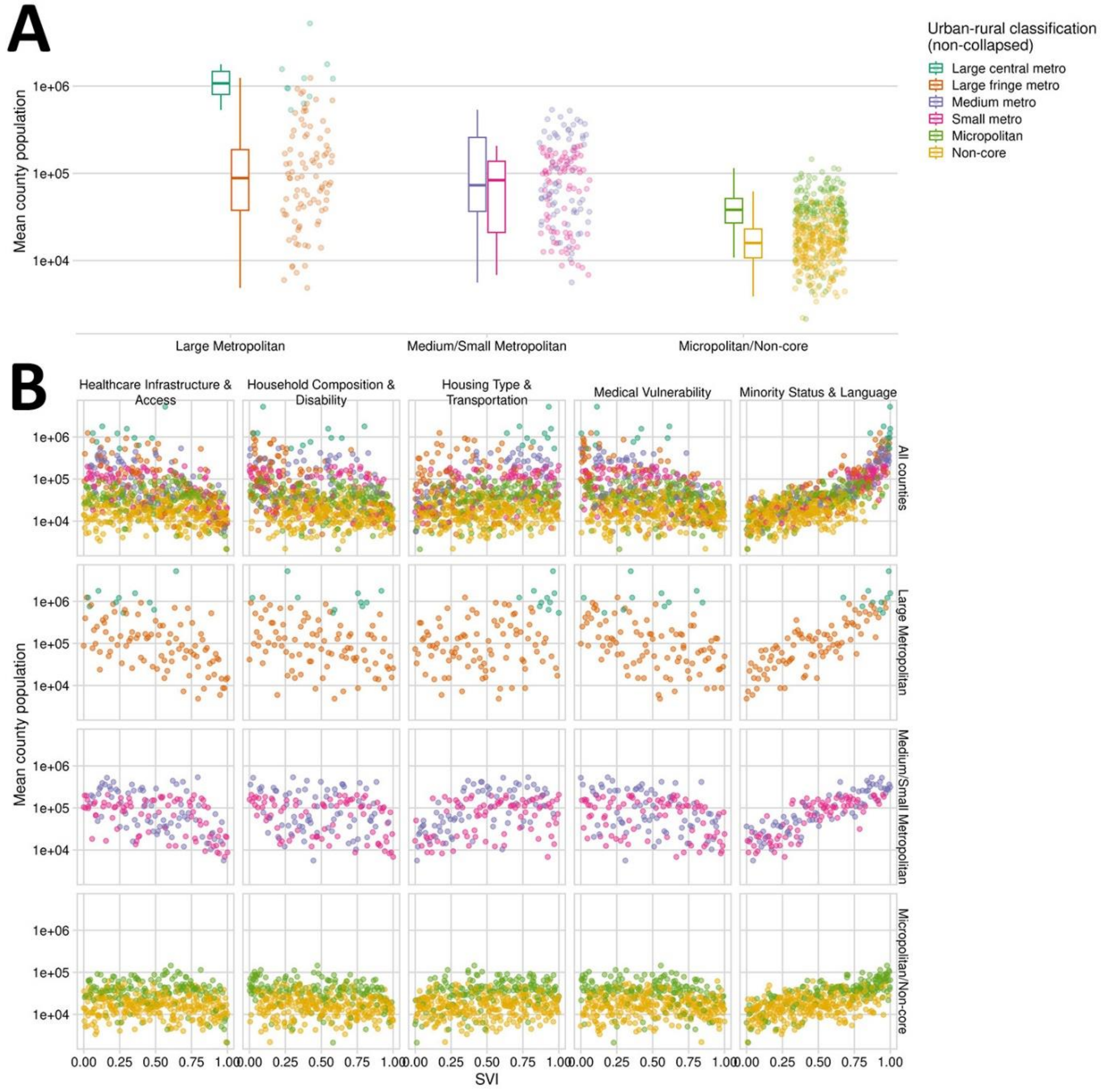

Appendix Figure 5. A) County urban-rural classification (collapsed, x-axis) vs. mean county population size. The boxplots show the median and interquartile range, and the points show the raw data. B) MH SVI vs. mean county population size (themes are columns and rows are the collapsed NCHS urban-rural classification). MH SVI theme scores are interpreted as percentiles; higher scores represent more vulnerable counties. The colors indicate the non-collapsed NCHS urban-rural classification. Population size (y-axis) is on a log scale.

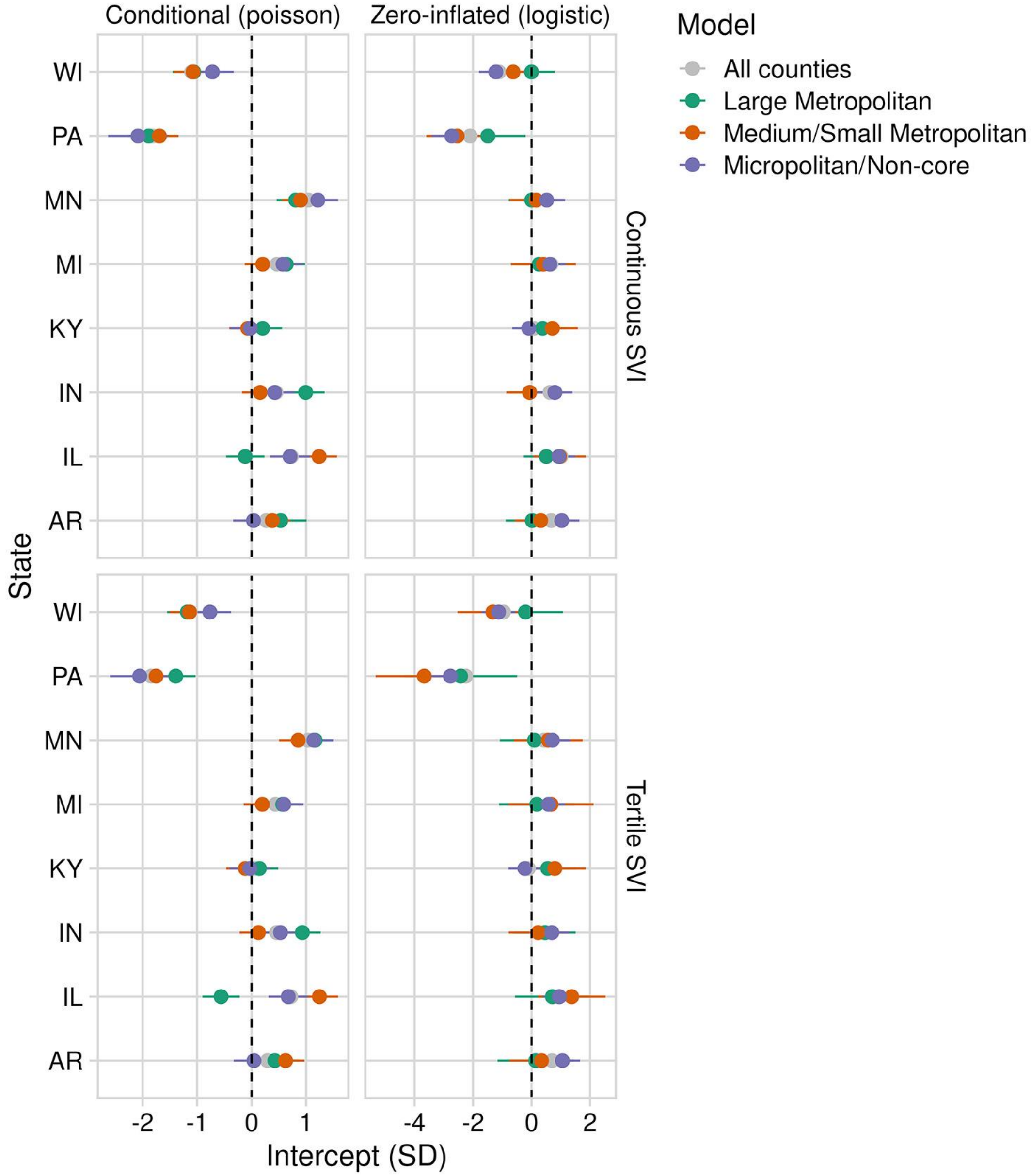

Appendix Figure 6. Estimated intercepts (and their standard deviations) for each state in the models. The row panels indicate how MH SVI themes were included in the model (either as continuous scores or tertiles), and the column panels are the conditional (predicting incidence) and the zero-inflated components (predicting the presence of at least one case) of the models. and color indicates the model (model with all counties vs. those stratified by urban-rural classification). MH SVI theme scores are interpreted as percentiles; higher scores represent more vulnerable counties.

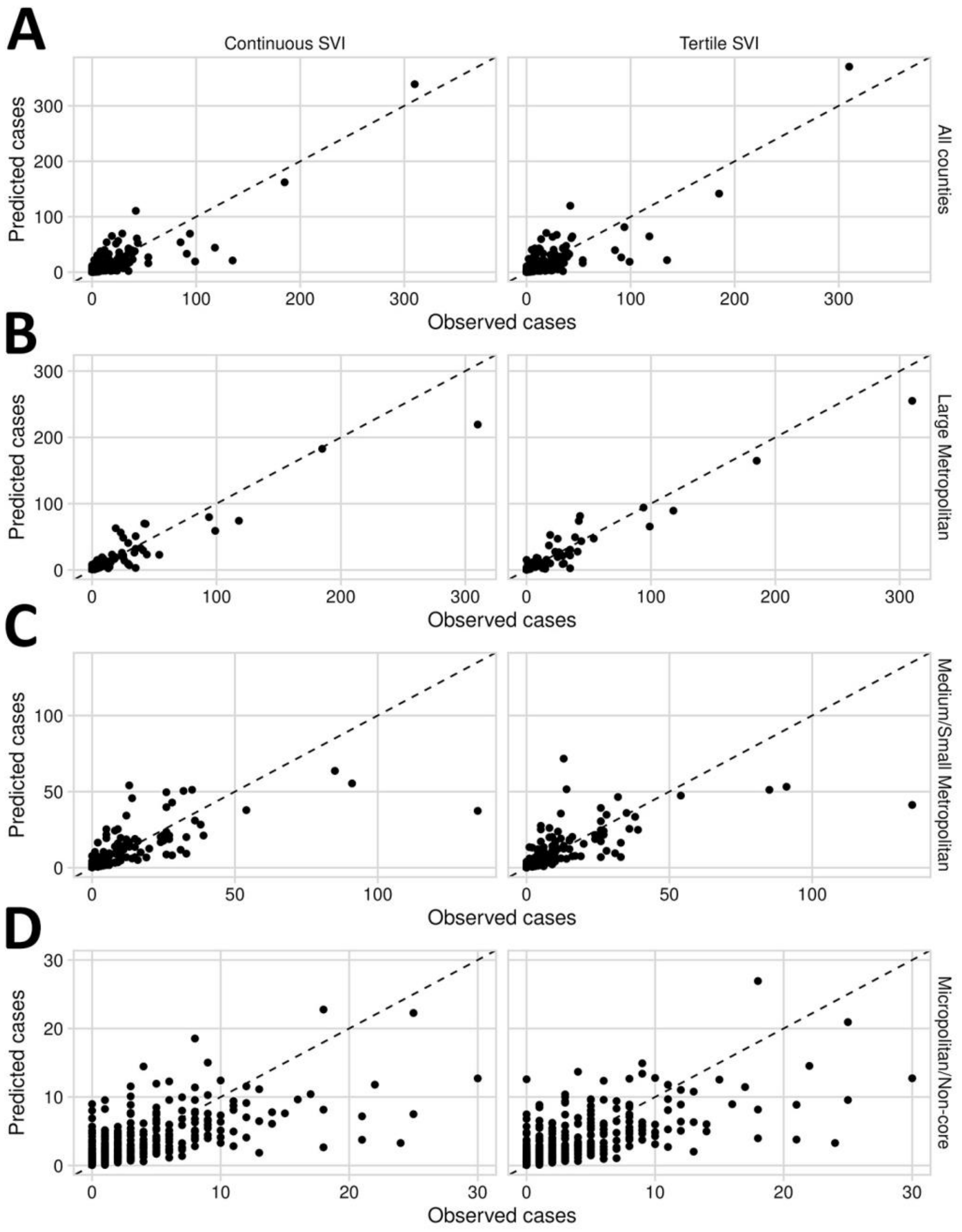

Appendix Figure 7. Observed vs. predicted cases for models fit to (A) all counties, (B) large metropolitan, (C) medium/small Metropolitan, and (D) micropolitan/non-core counties. The column panels are whether the model included MH SVI themes as a continuous covariate or as tertiles. The dashed line is the 1:1 prediction line, indicating a perfect concordance between predicted and observed values.

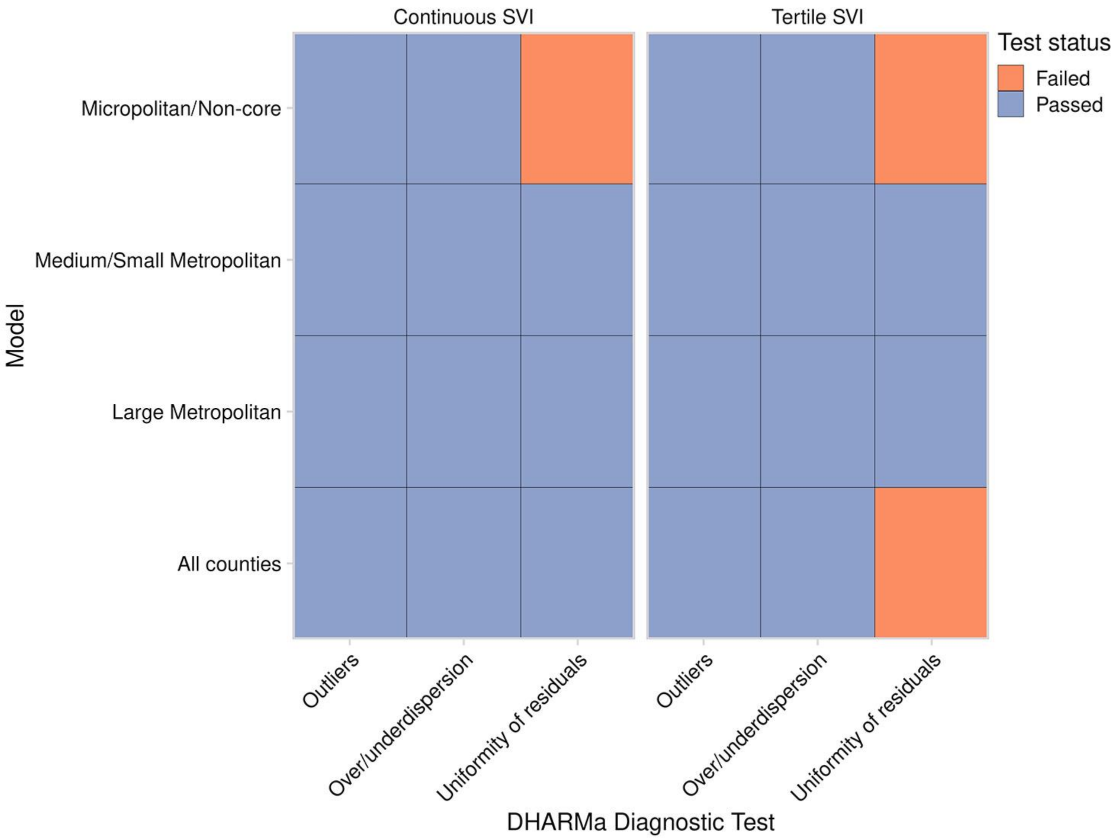

Appendix Figure 8. Model diagnostics with the diagnostic test type (see methods for more details) on the x-axis and the model type on the y-axis (all counties vs. models stratified by urban-rural classification). The color indicates whether the model passed the diagnostic test (tests failed when statistically significant at $p \leq 0.05$). The column panels indicate whether MH SVI themes were included as continuous covariates vs. tertiles.
